# Altered pattern in viral mRNA expression of Iranian type HTLV-1 leading to enhanced viral expression

**DOI:** 10.1186/1742-4690-11-S1-P146

**Published:** 2014-01-07

**Authors:** Takaharu Ueno, Runze Xun, Mineki Saito, Kenta Tezuka, Yuetsu Tanaka, Jun-ichi Fujisawa

**Affiliations:** 1 Department of Microbiology, Kansai Medical University, Hirakata, Osaka, Japan; 2 Department of Microbiology, Kawasaki Medical School, Kurashiki, Okayama, Japan; 3 Department of Immunology, Graduate School of Medicine, University of the Ryukyus, Nishihara, Okinawa, Japan

## 

Risk for HAM/TSP development is known to differ among the subtypes of HTLV-1. In Japan, incident rate of HAM/TSP in carrier people infected with cosmopolitan type A is 2.5 times higher than those with cosmopolitan type B. Although HTLV-1 of cosmopolitan type A is also prevalent in Jamaica and northern part of Iran, the rate of HAM/TSP development in this area is much higher than in Japan. Several polymorphisms in nucleotide sequence were found in pX region in Jamaican and Iranian type A compared to those in Japanese type A. These sequences include the substitution of stop codon to Trp codon, leading to extension of 20 a.a. in C-terminal of Rex. To assess the effect of these sequences on viral replication, we constructed the infectious clone harboring either Japanese type A (JP) or Jamaican/Iranian (J/I) pX sequences. Viral production from infectious clones in transiently transfected 293T cells was evaluated by p19 ELISA assay and J/I type virus was found to be produced higher than JP type. The virus produced in 293T cells was used to establish HTVL-1 infected Jurkat cell lines and viral mRNA expression was analyzed. In J/I type cell lines, full length mRNA was expressed more than doubly-spliced pX mRNA, whereas JP type cell lines exhibited the opposite pattern of mRNA expression. These results indicate that the higher viral production of J/I type should result from distinct post-transcriptional control most provably due to the difference in Rex protein. We are currently investigating the molecular mechanism of this phenomenon.

